# Longitudinal Evaluation of Cytopenias in the Renal Transplant Population

**DOI:** 10.1097/TXD.0000000000001339

**Published:** 2022-05-26

**Authors:** Aileen C. Johnson, Geeta Karadkhele, Wairimu Magua, Payas Vasanth, Christian P. Larsen

**Affiliations:** 1 Department of Surgery, Emory University, Atlanta, GA.; 2 Division of Nephrology, Department of Medicine, Emory University, Atlanta, GA.

## Abstract

Cytopenias, a common complication for immunosuppressed patients, are known to be associated with adverse transplant outcomes. However, there is little information on cytopenias in recipients treated with the costimulation blockade agent, belatacept.

**Methods.:**

We compared cytopenia incidence and manifestations in patients undergoing kidney transplant at Emory University Hospital on tacrolimus and belatacept. To reduce selection bias, the tacrolimus group was narrowed to include only patients eligible for belatacept.

**Results.:**

Of 1651 patients transplanted between 2009 and 2019, 187 (11%) experienced severe anemia, 309 (19%) experienced leukopenia, and 62 (4%) thrombocytopenia. On multivariable regressions, deceased-donor transplant, cytomegalovirus viremia, and thymoglobulin treatment were associated with risk of developing leukopenia, anemia, and thrombocytopenia. High-risk cytomegalovirus status was also associated with development of leukopenia and anemia. Additionally, azathioprine was associated with development of anemia, and both tacrolimus therapy and Caucasian race were associated with thrombocytopenia. Longitudinal quantifications of hematologic cell lines over the first-year posttransplant were extracted from generalized linear models fit using splines. Only hemoglobin range was significantly different between groups (greater in belatacept patients). Plots of mean cell count for each group suggest an earlier recovery from posttransplant anemia in belatacept patients.

**Conclusions.:**

Belatacept patients are not at increased risk of cytopenia but may have improved recovery from posttransplant anemia.

Over the past 2 decades, the transplant community has improved understanding of causes and outcomes associated with solitary cytopenias in kidney transplant recipients. However, an understanding of how this information can be used to tailor immunosuppression and improve clinical outcomes is still lacking. Longitudinal measures and robust characterization of cytopenias in the first-year posttransplant may be predictive of long-term outcomes and may vary by immunosuppression regimen. For instance, leukopenia and anemia are both listed as adverse reactions to belatacept by the manufacturer because *>*20% of patients experienced these cytopenias while on a belatacept regimen in clinical trials.^1^ However, minimal information is provided to clinicians about this relationship. As belatacept use in kidney transplant patients is slowly increasing and further applications to other organ transplant populations are explored, clinicians need access to information on patient outcomes in order to guide treatment decisions.^2-5^

In the postrenal transplant population, perturbations in cell counts are second only to kidney function tests in the impact they signify for a patient’s care.^6^ In particular, leukopenias can portend a variety of underlying causes and, therefore, create a therapeutic dilemma. Leukopenia is a relatively common complication after solid organ transplant with reports of 20% to 60% of patients experiencing some degree of leukopenia.^7-10^ This is most commonly attributed to T-cell depletion therapy or cytomegalovirus (CMV) viremia, although many patients never have a clear causative source identified.^6,9^ Other factors contributing to leukopenia are drug toxicities, often from antimetabolite medications such as mycophenolate mofetil or azathioprine, viral syndromes, autoimmune diseases such as lupus, immunodeficiency related to HIV, or benign preexisting leukopenic conditions that have been tied to ethnicity.^7,8^ Although it has been previously demonstrated that CD4 lymphopenia is associated with increased rates of graft failure, morbidity, and mortality after transplant,^11-13^ the predictive value of more routinely measured white blood cell (WBC) subsets has only recently been associated with inferior outcomes.^14^

Anemia represents another common affliction, with more than half of patients affected by the point of reaching stage V chronic kidney disease.^15^ Many patients with end-stage renal disease are already receiving erythropoietin injections for symptomatic anemia before transplantation, and the requirements for these injections have been linked to mortality in a dose-dependent fashion^16^ for the pretransplant population,^17-19^ although this has been posited to result from confounding-by-indication.^20^ Although the typical transplant patient will experience an increase in hemoglobin levels after transplantation,^21,22^ around 30% to 50% of patients will continue to suffer from posttransplant anemia (PTA).^23-26^ PTA is most commonly associated with impaired graft function but has also been related to deceased-donor transplant, antiviral medications, and immunosuppressants.^27,28^ In addition to graft function, PTA is a risk factor for mortality and graft loss^29,30^ as well as acute rejection.^31^ After results from the Correction of Hemogloblin and Outcomes in Renal Insufficiency^32^ and Cardiovascular Risk Reduction by Early Anemia Treatment with Epoetin Beta^33^ trials led to revised hemoglobin targets in patients with chronic kidney disease, consideration has been given to lower hemoglobin targets for kidney transplant recipients as well.^34^

Our tertiary, academic institution is a major center for renal transplants, performing >240 transplants/y over the study period. Our center adopted belatacept as standard of care in May 2012. Since then, belatacept has been used as primary immunosuppression in Epstein-Barr virus (EBV) seropositive recipients excepting April 2017 to November 2018 when the medication was not available for use in new transplant recipients. Review of Scientific Registry of Transplant Recipients data indicates our center has the largest post approval experience in the United States (not shown). The high frequency of belatacept use in our transplant population allows for comparison of outcomes between belatacept and calcineurin inhibitor-based therapy.^35,36^ We aim to first characterize single and multilineage cytopenias in our patient population and subsequently use longitudinal descriptors of cell count trajectories to examine the relationship of immunosuppression regimen with perturbations in hematologic cell counts.

## MATERIALS AND METHODS

### Patient Inclusion and Definitions

Adult patients undergoing kidney transplant at Emory University Hospital between the years 2009 and 2019 were included. Institutional review board approval was obtained before data collection. All patients underwent induction therapy with basiliximab and were then transitioned to maintenance regimens with mycophenolate mofetil, prednisone, and either belatacept or tacrolimus as the principal therapy. Patients on belatacept-based therapy regimens received a transient course of coimmunosuppression with tacrolimus as previously described.^36^ Beginning January 2016, tacrolimus targets were lowered to 5 to 8 ng/mL for the first 6 mo after surgery, 3 to 5 ng/mL for postoperative months 6 to 9 and weaned to off by month 12. Additionally, belatacept exposure was reduced to 10 mg/kg at transplant and 5 mg/kg mo thereafter. Patients on tacrolimus-based therapy had target levels 8 to 12 ng/mL for the first 6 mo after surgery and were lowered to 5 to 8 ng/mL thereafter. In order to reduce selection bias, patients receiving immunosuppression with tacrolimus were limited to those meeting center criteria for belatacept use. Specifically, only tacrolimus patients transplanted during times that belatacept was unavailable were included (January 2009–May 2012; April 2017–November 2018). EBV seronegative patients and patients with HIV were excluded. Study inclusion was not conditional on graft survival.

Leukopenia was defined as a WBC value <2000 cells/mm^3^. Clinically significant anemia was defined as hemoglobin <8 mg/dL. Thrombocytopenia was defined as platelets <75/mm^3^. To reduce noise from initial postprocedural complications, laboratory values were analyzed starting 60 d posttransplant through the end of the first postoperative year. CMV risk status was defined by donor and recipient immunoglobulin G status. CMV low-risk patients (donor and recipient seronegative) received antiviral prophylaxis with valacyclovir 1 g once a day for 3 mo. CMV moderate risk was defined as a seropositive recipient receiving an organ from a donor who was either seronegative or seropositive. Moderate risk recipients received antiviral prophylaxis with oral valganciclovir 450 mg daily for 3 mo posttransplant. CMV high risk was defined as a seronegative recipient who received an organ from a seropositive donor. High-risk recipients received antiviral prophylaxis with valganciclovir 450 mg daily for 6 mo posttransplant. CMV status was regularly assessed monthly throughout the first-year posttransplant, and in the case of viremia, biweekly until viral clearance.

### Statistical Analysis

Demographic and clinical variables were compared between immunosuppression groups using χ^2^ and Wilcoxon rank-sum test with a significance threshold of *P* < 0.05 (Table 1). Patients with severe leukopenia, anemia, thrombocytopenia, or pancytopenia (as defined by trough levels) were identified. Using the R stats package, univariable logistic regressions were performed with immunosuppression regimen as the variable of interest and each cytopenia (defined by trough) as the outcome. Next, multivariable logistic regressions were performed to adjust for demographic and clinical variables associated with cytopenias. Additional variables known to be associated with cytopenias were included in the multivariable model: age, race, gender, donor type, CMV risk status, treatment with thymoglobulin, treatment with azathioprine, and CMV viremia. Statistical analysis was performed using R version 4.0.3.

**TABLE 1.** Demographic and clinicopathologic features of patients undergoing kidney transplant at Emory University between 2009 and 2019, with χ^2^ analysis of the differing composition of belatacept vs tacrolimus-treated cohorts

Characteristic	Belatacept (n = 1059)^*a*^	Tacrolimus (n = 592)^*a*^	*P * ^*b*^
Age, y, median (IQR)	51 (42–61)	50 (41–59)	0.3
Male, n (%)	591 (56)	349 (59)	0.2
African American, n (%)	571 (54)	329 (56)	0.6
CMV risk category, n (%)			<0.01
High	142 (14)	77 (13)	
Moderate	761 (73)	458 (79)	
Low	144 (14)	45 (7.8)	
CMV viremia, n (%)	272 (26)	154(26)	>0.9
BK viremia (>10 000), n (%)	223 (21)	114 (19)	0.4
Other infections, n (%)	61 (5.8)	107 (18)	<0.01
Cause of ESRD, n (%)			0.5
HTN	289 (28)	171 (29)	
Diabetes	275 (26)	146 (25)	
PKD	110 (10)	49 (8.3)	
FSGS	93 (8.9)	46 (7.8)	
GN	52 (5.0)	29 (4.9)	
SLE	55 (5.2)	34 (5.7)	
Other	176 (17)	117 (20)	
Living donor, n (%)	410 (39)	191 (32)	0.01
Hospitalizations, mean (SD)	1.05 (1.58)	1.19 (1.72)	0.08
Thymoglobulin, n (%)	124 (12)	98 (17)	<0.01
Azathioprine, n (%)	26 (2.5)	10 (1.7)	0.4

^*a*^Statistics presented: median (IQR); n (%).

^*b*^Statistical tests performed: Wilcoxon rank-sum test; χ^2^ test of independence.

CMV, cytomegalovirus; ESRD, end-stage renal disease; FSGS, focal segmental glomerulosclerosis; GN, glomerulonephritis; HTN, hypertension; IQR, interquartile range; PKD, polycystic kidney disease; SLE, systemic lupus erythematosus.

### Longitudinal Summary Measures

Patterns in hematologic cell lines were tracked longitudinally by plotting WBC, platelets, and hemoglobin over time for each patient. Using the R bayestestR package, the area under the curve (AUC) was measured as the integral of P (the defined cutoff for each cytopenia: WBC = 2, hemoglobin = 8, and platelet = 75) − X (the cell count value at each point in time, with condition X = P, if X>P) with respect to time, over days 60 to 365 posttransplant. This value represents the amount of time the patient spent with a cell count below the threshold.


$$AUC=\;P-\int_{60}^{365}X\left(t\right)\;dt$$

Cell counts for each patient were fit with linear regression models using R stats core package. Models were fit with quintic spline functions (n = 5 *df*), using the R splines package to generate a B-spline basis matrix around the predictor variable (time). We use B-spline because this method is efficient for curve fitting and computationally easy to differentiate. The first derivative, or slope, of the fitted curve was calculated and the volatility of postoperative cell count was approximated as the mean of the absolute value of the slope of the curve. The range and trough of each cell line were also extracted.


$$Volatility=mean(\left|\frac{dx}{dt}\right|)$$

Cell counts were quantified using these methods: trough, range, volatility, and AUC were used as inputs for *t* test comparison to evaluate the difference in patterns of cytopenia between patients on belatacept and tacrolimus. In this context, the trough indicates the lowest cell count experienced by the patient, the range represents the difference between the lowest and highest value experienced by the patient, volatility indicates the propensity of a value to change, and AUC quantifies the magnitude of space bounded by cell count and time below a defined threshold. The mean and SD value of hemoglobin, WBCs, and platelets beginning at postoperative day 60 and extending to postoperative day 365 were calculated by immunosuppression regimen and plotted to visualize overall patterns between groups.

## RESULTS

### Patient Characteristics

After removing EBV seronegative patients, patients with HIV, and patients enrolled on tacrolimus during periods of belatacept availability, 1651 patients were included for analysis. Median age was 51 (42–61), and 57% of patients were male. Nine hundred patients (55%) identified as African American. Six hundred one (36%) received transplants from living donors, compared with 1050 (64%) who received transplants from deceased donors. One thousand fifty-nine patients received belatacept maintenance immunosuppression (64%), whereas 592 patients received tacrolimus (36%). Median follow-up was 4 y (1464 d). For each patient, there was on average 20 measurements of hemoglobin (SD: 19), 20 measurements of platelets (SD: 17), and 20 measurements of WBCs (SD: 17) between postoperative days 60 and 365. On χ^2^ analysis of baseline cohort demographic and clinical variables, patients on tacrolimus were more likely to be CMV moderate risk, to have received a transplant from a deceased donor, and to undergo therapy with thymoglobulin for treatment of acute rejection than those on belatacept (Table 1).

**FIGURE 1.**
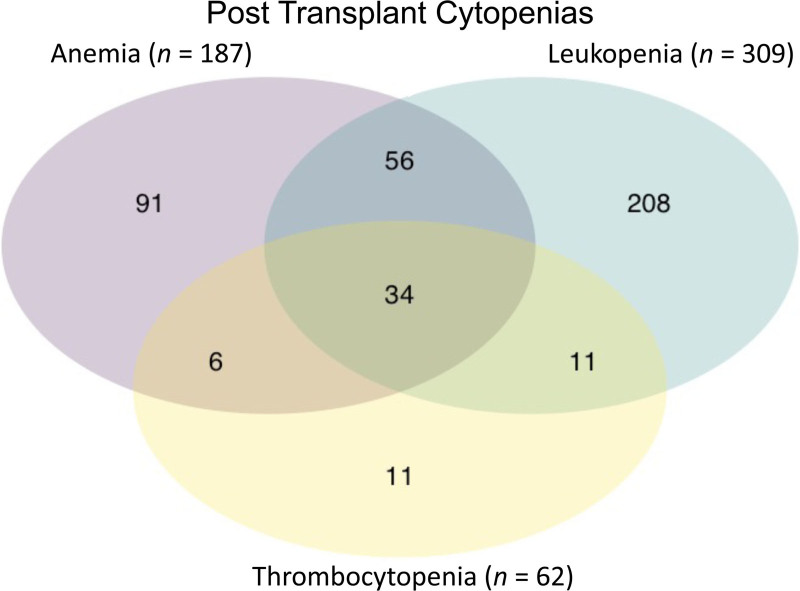
Venn diagram demonstrating the kidney transplant patients with anemia (hemoglobin <8 g/dL), leukopenia (<2 white blood cell [wbc]/microliter), and thrombocytopenia (<75 platelets/microliter) after initial 60 d posttransplant.

Cytopenias were very common in our population. Between postoperative days 60 and 365, 600 patients (36%) had a trough hemoglobin of <10, whereas 187 patients (11%) reached a trough hemoglobin <8, and 24 experienced a hemoglobin of <6 during this period. Eighty-six percent of anemic patients (161 of 187) received treatment with erythropoietin. Three hundred nine patients (19%) experienced leukopenia (minimum WBC <2) between postoperative days 60 and 365, and 105 of those (34%) had accompanying neutropenia (<500). One hundred sixty-three of 309 leukopenic patients (53%) were treated with granulocyte-colony stimulating factor (G-CSF). Neutropenic patients were treated at a higher rate, with 90% receiving G-CSF (94 out of 105). Patients on both immunosuppression regimens experienced decreasing rates of leukopenia over time parallel to reduction of immunosuppression. In the belatacept group, the incidence of leukopenia was 0.10% per day until 6 mo, 0.08% per day from 6 to 9 mo, and 0.06% per day from 9 to 12 mo. In the tacrolimus group, the rate was 0.13% per day until 6 mo, decreasing to 0.08% per day at 6 mo and 0.06% per day at 9 mo, similarly to the belatacept group. Only 4% of the entire study population (62 patients) experienced thrombocytopenia (platelets <75), and 34 patients (2%) exhibited pancytopenia (Figure 1).

### Factors Associated With Cytopenias

To explore the factors associated with cytopenias in our population, we performed both univariable and multivariable logistic regression for each cytopenia. First, we examined the factors associated with the outcome of leukopenia. On univariable analysis, tacrolimus immunosuppression was associated with increased odds of leukopenia (Table 2, odds ratio [OR] = 1.35, *P* = 0.02). However, this did not persist on multivariable regression, controlling for other clinical variables (**Table S1**, **SDC**, http://links.lww.com/TXD/A427). On multivariable analysis, patients with leukopenia were more likely to have received a kidney from a deceased donor (OR, 1.4, *P* = 0.03), to be CMV high risk (OR, 3.7, *P* < 0.01), to have undergone treatment of acute rejection with thymoglobulin (OR, 3.7, *P* < 0.01), or experienced CMV viremia (OR, 2.9, *P* < 0.01). A total of 52% of patients with severe leukopenia experienced CMV viremia, whereas 32% had undergone treatment of acute rejection with thymoglobulin. These 2 factors alone were present in 72% of patients who experienced leukopenia, and only 9% of patients who experienced leukopenia did not have at least 1 risk factor. Manual chart review was performed on patients for whom captured data did not offer an explanation for cytopenia. Leukopenias in this group were most often attributed to unknown viral syndromes, medication side effects, sepsis, or underlying medical conditions.

**TABLE 2.** Univariate regressions of cytopenias by immunosuppression regimen

Outcome	Odds ratio	95% confidence interval	*P*
Leukopenia	1.35	(1.04-1.73)	0.02
Anemia	1.20	(0.87-1.63)	0.26
Thrombocytopenia	1.71	(1.03-2.85)	0.04
Pancytopenia	2.31	(1.17-4.65)	0.02

Univariable regressions between immunosuppression regimen and each cytopenia. Immunosuppression regimen (tacrolimus) was associated with leukopenia, thrombocytopenia, and pancytopenia on univariable analysis but only thrombocytopenia and pancytopenia on multivariable regression (**Tables S4 through S7**, **SDC**, http://links.lww.com/TXD/A427). Belatacept was used as the reference group for this comparison.

Next, we examined the factors associated with PTA in our population. There was no association of anemia with tacrolimus versus belatacept immunosuppression on univariable analysis (Table 2). Multivariable analysis of anemia revealed that patients were more likely to be CMV high risk (OR, 2.2, *P* = 0.03), to have received a transplant from a deceased donor (OR, 1.8, *P* < 0.01), to be treated with azathioprine (OR, 2.3, *P* = 0.04), to have experienced CMV viremia (OR, 2.3, *P* < 0.01), and to have been treated with thymoglobulin (OR, 1.8, *P* < 0.01; **Table S2**, **SDC**, http://links.lww.com/TXD/A427).

The next step was analysis of factors associated with the outcome of thrombocytopenia. Univariable analysis revealed an increased odds of thrombocytopenia for patients on tacrolimus (Table 2, OR, 1.7, *P* = 0.04). On multivariable analysis of thrombocytopenia, tacrolimus immunosuppression was still associated with an increased odds (OR, 1.7, *P* = 0.04), as were deceased-donor transplant (OR, 3.4, *P* < 0.01), receipt of thymoglobulin (OR, 2.8, *P* < 0.01), and CMV viremia (OR, 3.8, *P* < 0.01). African American race was associated with a decreased odds of thrombocytopenia (OR, 0.5, *P* < 0.01; **Table S3**, **SDC**, http://links.lww.com/TXD/A427).

Finally, we examined the factors associated with the presence of pancytopenia in our patient population. Tacrolimus was associated with an increased odds of pancytopenia on univariable analysis (Table 2, OR, 2.3, *P* = 0.02). This increased odds ratio persisted on multivariable analysis (OR, 2.4, *P* = 0.02). Deceased-donor transplant (OR, 6.0, *P* < 0.01), treatment with thymoglobulin (OR, 3.3, *P* < 0.01), and CMV viremia (OR, 4.5, *P* < 0.01) were also associated with development of pancytopenia. Again, African American race was associated with a decreased odds of pancytopenia (OR, 0.3, *P* < 0.01; **Table S4**, **SDC**, http://links.lww.com/TXD/A427).

### Longitudinal Summary Measures

**FIGURE 2.**
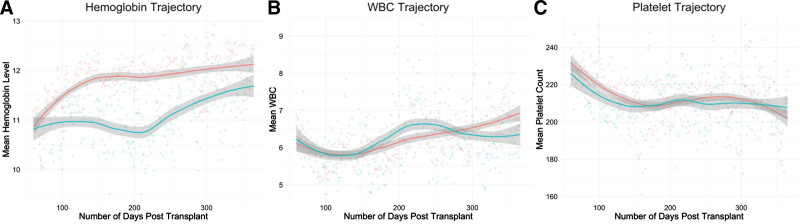
Longitudinal trajectories of belatacept patients (pink) compared with tacrolimus patients (blue) based on population average cell counts at each d posttransplant. Plots of hemoglobin (A), WBC (B), and platelet (C) levels over the first 365 d posttransplant are shown. Trajectories are similar between immunosuppression groups with the exception of greater hemoglobin increase seen in belatacept patients. WBC, white blood cell.

The trough, range, volatility, and AUC for each cytopenia were compared between immunosuppression regimens using 2-tailed Student *t* test with a significance threshold of *P* = 0.05 (Table 3). The hemoglobin range was significantly different between groups (Bela = 3.1, Tac = 2.9, *P* < 0.01), as was platelet range (Bela = 113, tac = 103, *P* = 0.02). Plots of mean cell counts for each immunosuppression group confirmed that increased hemoglobin range in belatacept patients corresponded to improved recovery from anemia (Figure 2). All other measures were not significantly different between groups, inclusion of laboratory measures out to 4 y posttransplant did not lead to the separation of any additional measures (data not shown).

**TABLE 3.** *T* test comparison of longitudinal features between belatacept and tacrolimus immunosuppression groups

Parameter	Belatacept^*a*^	Tacrolimus^*a*^	*P*
Hemoglobin AUC	3.20 (10.68)	2.85 (10.75)	0.53
Hemoglobin range	3.15 (1.56)^*b*^	2.94 (1.62)^*b*^	<0.01^*b*^
Hemoglobin trough	10.7 (1.90)	10.5 (2.04)	0.30
Hemoglobin volatility	0.05 (0.54)	0.27 (3.91)	0.17
Platelet AUC	33.29 (721.19)	32.95 (443.87)	0.99
Platelet range	114.10 (73.63)	107.47 (84.45)	0.11
Platelet trough	171.19 (54.84)	168.37 (59.07)	0.35
Platelet volatility	2.20 (25.03)	5.73 (58.44)	0.17
WBC AUC	3.08 (15.15)	3.27 (11.46)	0.77
WBC range	5.90 (4.30)	5.67 (4.27)	0.30
WBC trough	3.91 (1.96)	3.76 (2.02)	0.15
WBC volatility	0.08 (0.54)	0.81 (11.44)	0.12

^*a*^Statistics presented: mean (SD).

^*b*^ Statistical significance.

AUC, area under the curve; WBC, white blood cell.

## DISCUSSION

Although analysis methods of cytopenias vary across the literature, the burden of evidence supports the clinical weight of, in particular, leukopenia and anemia towards transplant patient outcomes.^14,29-31^ To our knowledge, this study represents the first evaluation of the 3 hematologic cell lines in conjunction. We chose to use relatively strict definitions for each cytopenia and include only laboratory values after postoperative day 60 in order to eliminate noise from surgical complications as well as changes that were not clinically significant. Further more, we extracted multiple summary measures of hematologic cell line trajectories in order to more rigorously capture differences between cohorts.

This is also the first study to focus on cytopenias in a large cohort of kidney transplant recipients receiving belatacept immunosuppression, addressing a crucial gap in the field. Although the package insert for belatacept lists both leukopenia and anemia as common adverse reactions to the medication, rates of cytopenias were similar in comparison to the control group, cyclosporine (20% versus 23% leukopenia, 45% versus 44% anemia).^1^ However, this comparison has not previously been extended to modern tacrolimus-based regimens. Our analysis demonstrated that immunosuppression regimen (tacrolimus based versus belatacept based) was only minimally associated with any longitudinal trends in hematologic cell lines. Although cytopenias are common in the transplant population, belatacept patients do not appear to be at any increased risk. In particular, neither immunosuppression regimen was found to be associated with leukopenia on multivariate regression. The only clinically relevant difference detected between immunosuppression regimens was belatacept patients’ increased average hemoglobin range, which may be associated with the increase in average estimated glomerular filtration rate posttransplant seen in belatacept patients compared with patients on calcineurin inhibitors.^36,37^ On logistic regression, patients on tacrolimus immunosuppression were also more likely to develop thrombocytopenia and pancytopenia, which persisted on multivariable regression. Although it is possible this is related to residual selection bias between immunosuppression regimens, we have attempted to exclude tacrolimus-treated patients who would not be eligible for belatacept immunosuppression. Differences between baseline demographics of the 2 groups (ie, rate of living donor transplant) may be due to persistent latent selection bias or changes in practice patterns over time.

Regression analysis in our population was consistent with prior studies in identifying thymoglobulin, CMV viremia, and deceased-donor transplant as risk factors with high odds of cytopenia development across hematologic cell lines. In our cohort, a high percentage of anemic and neutropenic patients are receiving appropriate therapy with erythropoietin or G-CSF. The small group of leukopenic patients that did not experience at least 1 expected risk factor presents a cohort of interest for future studies, as many presented with symptoms consistent with viral syndromes without a positive diagnostic test.

In the current era of electronic medical records, high-density data on objective measures such as laboratory values are easily available. However, methods to include analysis of this data are only slowly being incorporated into studies of clinical outcomes. Clinical laboratory values play a major role in patient care decisions; however, capturing a static, individual measurement is unlikely to accurately portray a patient’s clinical scenario. The experienced clinician has developed an almost subconscious pattern recognition that informs medical decision making. Frequently, however, studies considering measured laboratory values as prognostic markers in the transplant population do not adequately incorporate longitudinal measures that allow consideration of a patient’s baseline or overall trajectory, but more commonly rely on reductionist measurements such as peaks or troughs. Model fitting and extraction of descriptive metrics provide an alternative to using time-dependent covariates in analyzing patient outcomes. Future studies should consider using clustering of laboratory value trajectories as a means to preserve multiple measures of value trajectories while reducing the dimensions for analysis.

Although this information is important for clinicians prescribing immunosuppression, this study does have a number of limitations. This is a single-center retrospective study, and despite attempts to reduce differences between immunosuppression cohorts, is still likely subject to residual selection bias. Laboratory measurements were drawn as clinically indicated, resulting in a higher density of samples for patients with active pathology, which may affect the findings of this study. Additionally, due to strict thresholds for cytopenia definitions, some cohorts were relatively small (pancytopenia and thrombocytopenia), which creates the risk of overfitting in multivariable logistic regression modeling. Conclusions drawn from analysis of these small cohorts should be interpreted with caution.

In conclusion, we have demonstrated that patients on belatacept are not at increased risk of cytopenia. Patients on belatacept therapy appear to have a greater increase in hemoglobin levels postoperatively, most likely related to a greater improvement in estimated glomerular filtration rate. In addition, patients on tacrolimus immunosuppression are at higher risk for thrombocytopenia and pancytopenia. The use of time series analysis to capture longitudinal patterns of laboratory values merits further exploration.

## Supplementary Material


