# Supplementary material for: Longitudinal Evaluation of Cytopenias in the Renal Transplant Population
Source: Transplant Direct. 2022 May 26;8(6):e1339. doi: 10.1097/TXD.0000000000001339 (PMC9148693; doi:10.1097/TXD.0000000000001339)
Supplement: Supplementary file 1 [file txd-8-e1339-s001.pdf]

Table S1.

Multivariable Regression: Outcome Leukopenia			
**Parameter**	Odds Ratio	95% Confidence Interval (CI)	p-value
**CMV Risk: High**	3.71	[2.02, 7.20]	<0.01
**CMV Risk: Moderate**	1.48	[0.86, 2.74]	0.18
**Immunosuppression: Tacrolimus**	1.30	[0.98, 1.71]	0.07
**Donor Type: Deceased**	1.41	[1.04, 1.93]	0.03
**Azathioprine**	1.53	[0.66, 3.32]	0.29
**Age at time of transplant, years**	1.01	[1.00, 1.02]	0.18
**Race: African-American**	0.87	[0.65, 1.16]	0.33
**Thymoglobulin**	3.66	[2.61, 5.14]	<0.01
**CMV Viremia**	2.88	[2.16, 3.85]	<0.01

Table S1. Multivariable regression examining clinical variables association with leukopenia. High-risk CMV status, deceased donor, thymoglobulin treatment, and CMV viremia were associated with leukopenia.

Table S2

Multivariable Regression: Outcome Anemia			
**Parameter**	Odds Ratio	95% Confidence Interval (CI)	p-value
**CMV Risk: High**	2.19	[1.08, 4.75]	0.04
**CMV Risk: Moderate**	1.16	[0.62, 2.36]	0.66
**Immunosuppression: Tacrolimus**	1.16	[0.84, 1.61]	0.36
**Donor Type: Deceased**	1.85	[1.27, 2.74]	<0.01
**Azathioprine**	2.28	[0.97, 4.92]	0.04
**Age at time of transplant, years**	1.01	[1.00, 1.03]	0.05
**Race: African-American**	1.22	[0.87, 1.73]	0.26
**Thymoglobulin**	1.78	[1.17, 2.67]	0.01
**CMV Viremia**	2.29	[1.62, 3.23]	<0.01

Table S2. Multivariable regression examining clinical variables association with anemia. High-risk CMV status, deceased donor, azathioprine treatment, thymoglobulin treatment, and CMV viremia were associated with anemia.

Table S3

Multivariable Regression: Outcome Thrombocytopenia			
**Parameter**	Odds Ratio	95% Confidence Interval (CI)	p-value
**CMV Risk: High**	1.38	[0.49, 4.55]	0.57
**CMV Risk: Moderate**	0.55	[0.21, 1.70]	0.25
**Immunosuppression: Tacrolimus**	1.74	[1.02, 2.97]	0.04
**Donor Type: Deceased**	3.36	[1.72, 7.11]	<0.01
**Azathioprine**	2.08	[0.46, 6.60]	0.27
**Age at time of transplant, years**	1.01	[0.99, 1.03]	0.5
**Race: African-American**	0.54	[0.31, 0.94]	0.03
**Thymoglobulin**	2.71	[1.45, 4.94]	<0.01
**CMV Viremia**	3.76	[2.10, 6.86]	<0.01

Table S3. Multivariable regression examining clinical variables association with thrombocytopenia. Tacrolimus immunosuppression, deceased donor transplant, thymoglobulin treatment, and CMV viremia were associated with thrombocytopenia, while patients who identified as black had a lower risk of thrombocytopenia.

Table S4

Multivariable Regression: Outcome Pancytopenia			
**Parameter**	Odds Ratio	95% Confidence Interval (CI)	p-value
**CMV Risk: High**	6.68×10^{6}	[1.14×10^{-9}, 5.05×10^{99}]	0.98
**CMV Risk: Moderate**	1.95×10^{6}	[1.63×10^{-8}, 2.68×10^{108}]	0.98
**Immunosuppression: Tacrolimus**	2.36	[1.15, 4.93]	0.02
**Donor Type: Deceased**	5.98	[2.20, 21.10]	<0.01
**Azathioprine**	1.01	[0.05, 5.82]	0.99
**Age at time of transplant, years**	1.01	[0.98, 1.04]	0.55
**Race: African-American**	0.34	[0.16, 0.72]	0.01
**Thymoglobulin**	3.25	[1.42, 7.24]	<0.01
**CMV Viremia**	4.51	[2.08, 10.42]	<0.01

Table S4. Multivariable regression examining clinical variables association with pancytopenia. Treatment with tacrolimus, deceased donor transplant, thymoglobulin treatment, and CMV viremia were associated with thrombocytopenia, while patients who identified as black had a lower risk of pancytopenia.
